# Nicotinamide Suppresses Hyperactivation of Dendritic Cells to Control Autoimmune Disease through PARP Dependent Signaling

**DOI:** 10.3390/nu16162665

**Published:** 2024-08-12

**Authors:** Ai-Ping Cao, Yun-Ying Wang, Yan-Yan Shen, Yan-Hong Liu, Jia-Yu Liu, Yao Wang, Yue Guo, Rui-Bo Wang, Bo-Yang Xie, Xin Pan, Ai-Ling Li, Qing Xia, Wei-Na Zhang, Tao Zhou

**Affiliations:** 1 Institute of Translational Medicine, Zhejiang University School of Medicine, Hangzhou 310016, China; 12118135@zju.edu.cn (A.-P.C.); alli@ncba.ac.cn (A.-L.L.); 2 Nanhu Laboratory, National Center of Biomedical Analysis, Beijing 100039, China; 3170103695@zju.edu.cn (Y.-Y.W.); 18997269090@163.com (Y.-Y.S.); lyhyjyy@163.com (Y.-H.L.); liujiayu6066@163.com (J.-Y.L.); ywang@xmail.ncba.ac.cn (Y.W.); guoyue8899@163.com (Y.G.); 18810622983@163.com (R.-B.W.); xby19990318@163.com (B.-Y.X.); xpan@ncba.ac.cn (X.P.); qxia@ncba.ac.cn (Q.X.)

**Keywords:** dendritic cell, nicotinamide, PARP, NF-κB, psoriasis

## Abstract

Dendritic cells (DCs) are crucial in initiating and shaping both innate and adaptive immune responses. Clinical studies and experimental models have highlighted their significant involvement in various autoimmune diseases, positioning them as promising therapeutic targets. Nicotinamide (NAM), a form of vitamin B3, with its anti-inflammatory properties, has been suggested, while the involvement of NAM in DCs regulation remains elusive. Here, through analyzing publicly available databases, we observe substantial alterations in NAM levels and NAM metabolic pathways during DCs activation. Furthermore, we discover that NAM, but not Nicotinamide Mononucleotide (NMN), significantly inhibits DCs over-activation in vitro and in vivo. The suppression of DCs hyperactivation effectively alleviates symptoms of psoriasis. Mechanistically, NAM impairs DCs activation through a Poly (ADP-ribose) polymerases (PARPs)-NF-κB dependent manner. Notably, phosphoribosyl transferase (NAMPT) and PARPs are significantly upregulated in lipopolysaccharide (LPS)-stimulated DCs and psoriasis patients; elevated NAMPT and PARPs expression in psoriasis patients correlates with higher psoriasis area and severity index (PASI) scores. In summary, our findings underscore the pivotal role of NAM in modulating DCs functions and autoimmune disorders. Targeting the NAMPT-PARP axis emerges as a promising therapeutic approach for DC-related diseases.

## 1. Introduction

DCs serve as crucial mediators between innate and adaptive immunity. Upon encountering pathogens or inflammatory signals, DCs undergo maturation and activation, primarily through the NF-κB pathway. This activation leads to the induction of costimulatory factors such as CD80, CD86, major histocompatibility complex (MHC) molecules, and inflammatory cytokines, further promoting the maturation and antigen-presenting capabilities of DCs. Subsequently, activated DCs stimulate T cell immune responses, facilitating the orchestration of the adaptive immune system [1,2]. However, abnormal activation of DCs can lead to various diseases. Insufficient activation of DCs weakens the immune system, rendering the body more susceptible to pathogen infections or tumor attacks, whereas excessive activation can lead to inflammatory damage or autoimmune diseases [3]. Therefore, it is essential to target DCs and regulate their activation in the context of disease. Several studies have demonstrated the potential for boosting immunity by modulating DCs through genetic, protein, or metabolic agents in the context of tumor and viral infections, leading to successful therapeutic outcomes in clinical practice [4,5]. Nevertheless, the regulatory mechanisms of DCs in autoimmune diseases remain unclear, with clinical therapeutic effects often being suboptimal.

Psoriasis is a chronic inflammatory skin disorder, with its immune mechanisms extensively elucidated in recent years [6]. Psoriasis is characterized by epidermal hyperplasia and the infiltration of immune cells into the dermis [7]. In psoriasis, the activation of plasmacytoid dendritic cells (pDCs) promotes the maturation of myeloid dendritic cells (mDCs), which are responsible for presenting antigens to T cells. This antigen presentation activates T cells to produce a cascade of pro-inflammatory cytokines, such as IL-17 and IL-22, which leads to hyperproliferation of keratinocytes and the formation of psoriatic plaques [8]. Additionally, DCs in psoriasis produce high levels of cytokines like TNF-α, IL-23, and IL-12, amplifying the inflammatory response and contributing to the persistence of the disease [1,9]. While several anti-psoriatic drugs, particularly biologics, have shown promising efficacy, long-term effectiveness and safety remain significant challenges in psoriasis treatment. Thus, there is an urgent need to explore more efficient treatments for psoriasis therapy, especially targeting DCs [10].

NAM, a form of vitamin B3 (niacin), serves as a precursor for the coenzymes nicotinamide adenine dinucleotide (NAD^+^), with NAMPT catalyzing the initial reversible step in NAD^+^ biosynthesis. NAM was originally used to treat pellagra and operates by elevating NAD^+^ levels [11,12]. Recent research has found that NAM, as a precursor for NAD^+^, can enhance the efficacy of tumor immunotherapy by promoting PD-L1 expression and boosting the anti-tumor activity of natural killer cells [13,14]. Additionally, it has been discovered that NAM exhibits anti-inflammatory properties and effectively alleviates collagen-induced arthritis symptoms in mice when used together with methotrexate [15,16]. Thus far, research on NAM itself has been limited, particularly regarding its specific mechanisms in psoriasis and its potential role in modulating immune cells during disease progression.

In this study, we identified NAM as a natural suppressor of DCs function, characterized by its level changes during DCs activation. Subsequent research demonstrated that NAM significantly suppressed DCs activation in vitro and restrained DCs infiltration, mitigating psoriasis symptoms in mouse models. Analysis of clinical psoriasis databases further indicated a positive correlation between NAMPT overexpression and DCs hyperactivation, consistent with our observation that reduced NAM levels contribute to heightened DCs activation. Mechanistically, NAM inhibited DCs activation by serving as a PARP inhibitor, thereby impeding the NF-κB signaling pathway. Importantly, we found that the NAMPT-PARP axis emerged as a potential predictor of disease severity. Collectively, our study underscored NAM’s therapeutic potential in autoimmune diseases beyond its role in NAD^+^ biosynthesis, emphasizing its direct impact on DCs mediated immune regulation and disease pathogenesis mechanisms.

## 2. Materials and Methods

### 2.1. Cell Isolation and Culture

For BMDCs preparation, bone marrow cells were flushed from mouse tibias and femurs, and cells were then plated in RPMI 1640 medium supplemented with 10% (*v*/*v*) FBS, 1% (*v*/*v*) penicillin-streptomycin, 50 μM β-mercaptoethanol, recombinant mouse GM-CSF (10 ng/mL; Peprotech, Cranbury, NJ, USA) and IL-4 (5 ng/mL; Peprotech). The cell medium was replaced with RPMI 1640 containing GM-CSF and IL-4 on day 3, and immature DCs were collected on day 6. To generate mature DCs, the immature DCs were stimulated with LPS (1 μg/mL; L2630, Sigma-Aldrich, St. Louis, MO, USA) for 24 h at 37 °C with 5% CO_2_.

For the isolation of OT-II cells, OT-II mice [C57BL/6-Tg (Tcra Tcrb) 425Cbn] were utilized. Spleens from OT-II mice were harvested in PBS (10010023; Gibco, Waltham, MA, USA) and mashed through a 70 μm cell strainer. Naïve T cells were enriched using the CD4^+^ T Cell Isolation Kit (130-104-453; Miltenyi Biotec, Bergisch Gladbach, Germany) according to the manufacturer’s instructions and were subsequently sorted as CD45^+^CD3^+^CD4^+^ cells.

### 2.2. DC Stimulation

Immature BMDCs were treated for 24 h with 1 μg/mL LPS. For the in vitro antigen presentation assay, immature BMDCs at day 6 were treated with EαGFP peptide (50 μg/mL) for 24 h at 37 °C with 5% CO_2_. Antigen uptake and presentation were assessed by flow cytometry. BMDCs were pretreated by 10 mM NAM (N0636, Sigma-Aldrich), 500 μM NMN (HY-F0004, MedChem Express, Shanghai, China) or 5 μM Venadaparib (IDX-1197) (HY-137457, MedChem Express) for 2 h when needed.

### 2.3. In Vitro T Cell Activation Assays

For in vitro co-culture assays, 4 × 10^4^ immature BMDCs were treated with 1 μg/mL LPS for 12~18 h and pulsed with 10 μg/mL OVA_323–339_ (T510211, Sangon, Shanghai, China) for 2 h, then the cells were washed twice with preheated RPMI 1640 culture medium. Subsequently, treated BMDCs and CFSE (C34570, Thermo Fisher, Waltham, MA, USA)-labeled naive OT-II cells were co-cultured in a 96-well plate for 72 h at a 1:5 DCs/T cells ratio (4 × 10^4^ DCs/2 × 10^5^ T cells). The proliferation and activation of OT-II cells were measured by flow cytometry analysis. BMDCs were pretreated by NAM or NMN for 2 h when needed.

### 2.4. Isolation of Mouse Skin and Draining Lymph Node (DLN) Cells

The isolation method of mouse skin cells was described previously [17]. In brief, mouse back skin was separated at the indicated time point, cut into small pieces and incubated in RPMI 1640 medium containing 1 mg/mL collagenase IV (C5138; Sigma-Aldrich), 50 μg/mL DNase I (A610099; Sangon), 10 mM HEPES (E607018; Sangon) and 10% FBS (12657-029; Gbico) at 37 °C for 90 min. Digested skin pieces were filtered through a 70 μm nylon mesh, and the suspensions were supplemented with RPMI 1640 to inactivate enzyme activity. The supernatant was then removed by centrifugation, and cell pellets were resuspended in PBS for cell counting.

For isolation of DLN cells, DLN from mice was extracted, grounded, passed through a 70 μm nylon mesh, and centrifuged at 1500 rpm for 5 min. Cell pellets were carried out, and cell counting was performed after re-selection with PBS.

### 2.5. Generation of Eα-EGFP Fusion Protein

Two DNA oligomers encoding the sense and antisense sequences of the Eα chain (amino acids 56–73) from the I-E^d^ allele were synthesized (Tsingke Biotechnology, Beijing, China) and annealed [18]. The double-stranded fragment was inserted in the pEGFP-N1, and the fragment containing the Eα-EGFP sequences was amplified by PCR and inserted into the pET28a expression vector. Then, the His-tagged EαGFP was prepared and purified using previously described methods [19]. The concentrated protein was collected, filtered through a 0.22 μm syringe filter, and stored at −80 °C until use.

### 2.6. Mice

The research conducted in this study complies with all of the relevant ethical regulations. C57BL/6 mice were obtained from Beijing Vital River Laboratory Animal Technology (Beijing, China) (stock: 219). OT-II mice were bought from Shanghai model Organisms (Shanghai, China) (stock: 004194). All mice were housed in the specific pathogen-free environment at 21 ± 1 °C and 60 ± 5% humidity, with a 12-h light/dark cycle. Experimental and control animals were bred separately. All mice were used at 6–10 weeks old and euthanized with carbon dioxide. All animal studies were performed in compliance with all relevant ethical regulations and were approved by the Institutional Animal Care and Use Committee (IACUC) at the Laboratory Animal Center of NANHU Laboratory.

To obtain bone marrow and spleen cells, we used male mice aged 6–10 weeks with a C57BL/6 background.

For the IMQ-induced mouse model of psoriasis, female C57BL/6 mice (7~8 weeks of age) were maintained under SPF conditions. The mice were topically treated with 62.5 mg of commercially available 5% IMQ cream (120503, Med-shine Pharma, Chengdu, China) on their shaved backs for six consecutive days [20]. The severity of back skin was assessed by thickness, scaling, and erythema daily according to the clinical Psoriasis Area and Severity Index. The back skin thickness was measured using a vernier caliper. Scaling and erythema were scored from 0 to 4: 0, none; 1, slight; 2, moderate; 3, marked; and 4, very marked. On day 6, mice were euthanized, and samples were harvested for subsequent experiments. For NAM treatment, the mice were exposed to NAM (300 mg/kg per mouse; N0636, Sigma-Aldrich) via intraperitoneal injection daily.

### 2.7. Flow Cytometry

For flow cytometry examination, all the cells for surface marker analysis were incubated with MACS buffer (130-091-221, Miltenyi Biotec) on ice for 10 min and then stained with the indicated antibodies on ice for 30 min in a dark place. Cell viability was measured by 7-Amino-Actinomycin D (7-AAD, 559925, BD Biosciences, Milpitas, CA, USA). Antibodies used in this study are as following: The mature phenotypes of BMDCs were stained with APC-anti-CD11c (117310, Biolegend, San Diego, CA, USA), PE-anti-MHC II (11-5321-81, Thermo Fisher), FITC- anti-CD86 (105006, Biolegend), PE-anti-CD80 (104708, Biolegend) and FITC-anti-MHC I (114606, Biolegend); For EαGFP antigen presentation assay, the BMDCs were stained with biotin-conjugated anti-mouse Eα 52–68 peptide (13-5741-81, eBioscience, San Diego, CA, USA), then washed twice with MACS buffer and stained with APC-conjugated streptavidin (405207, Biolegend); DCs in DLN and skin were stained with Pacific Blue-anti-CD45 (103126, Biolegend), APC-anti-CD11c, PE-anti-MHC II, FITC-anti-CD86, PE-anti-CD80 and FITC-anti-MHC I; The activation of OT II cells were stained with PE-CD4 and then fixed with 4% paraformaldehyde (PFA, P1110, macgene, Beijing, China) at room temperature (RT) for 15 min and followed by staining with PE-anti-IFN-γ, PE/Cy7-anti-IL17A and PE-anti-IL4 using Perm/Wash Buffer (554723, BD Biosciences) according to the manufacturer’s protocol (Detailed information about antibodies are provided in Supplementary Tables S1 and S2).

Flow cytometry data were acquired on LSR Fortessa (BD Biosciences) using BD FACSDiva software (v8) and analyzed using FlowJo software (v10) (Tree Star, Ashland, OR, USA). 

### 2.8. ELISA

Culture supernatant from in vitro antigen presentation assays was collected, and the levels of IFNγ, IL4, and IL17A were determined using IFNγ (430804), IL4 (431104), and IL17A (432504) (all purchased from Biolegend) ELISA kits according to manufacturer’s instructions.

### 2.9. RNA Isolation and Gene Expression Profiling

Total RNA was isolated from cells or skin tissues with Trizol reagent (T3934, Sigma-Aldrich), and cDNA was transcribed using PrimeScript RT Master Mix (RR036A, Takara, Shiga, Japan). qRT-PCR analysis was performed using the PowerUpN SYBRN Green (A25742, Thermo Fisher) method on the QuantStudio3 real-time fluorescence PCR instrument (Thermo Fisher Applied Biosystems, Waltham, MA, USA). The gene expression was normalized to the expression of the gene encoding Gapdh. Sequences of the primers for qRT-PCR were shown in Supplementary Table S3.

### 2.10. Gene Set Enrichment Analysis (GSEA)

GSEA was performed using the GSEA software [21] (version 4.3.2), the Reactome/BioCarta/chemical and genetic perturbations (CGP) subset of the Molecular Signature Database [22] (https://www.gsea-msigdb.org/gsea/index.jsp, accessed on 1 June 2024). One thousand total permutations were used. The terms with *p*-values < 0.05 were considered to be significantly enriched.

To estimate the infiltration levels of various immune cell types in each sample, ssGSEA was performed using the GSVA R package [23] (version 1.50.5) to calculate an enrichment score for each gene set in each sample, allowing for the quantification of immune cell infiltration levels based on predefined immune cell marker gene sets [24].

### 2.11. Differentially Expressed Genes Analysis

Differential expression analysis of two conditions/groups was performed using the Bioconductor-DESeq2 R package [25] (version 1.32.0) or Bioconductor-Limma R package [26] (version 3.40.6). Genes with fold change >2 or <0.5 and *p*-value < 0.05 were defined as differentially expressed.

### 2.12. Correlation Analysis

Spearman correlation coefficients were calculated to assess the relationships between the expression levels of different genes within the normalized matrix, as well as between the expression levels of each gene and clinical parameters. The significance of these correlations was determined using *p*-values, with thresholds set as follows to denote varying levels of statistical significance: * *p* < 0.05, ** *p* < 0.01, *** *p* < 0.001, and **** *p* < 0.0001.

### 2.13. Statistical Analysis

Statistical analyses were performed with GraphPad Prism software v9. Results were presented as the mean ± SD. A value of *p* < 0.05 was considered to indicate statistical significance.

## 3. Results

### 3.1. NAM, but Not NMN, Inhibits DCs Activation

**Figure 1:**
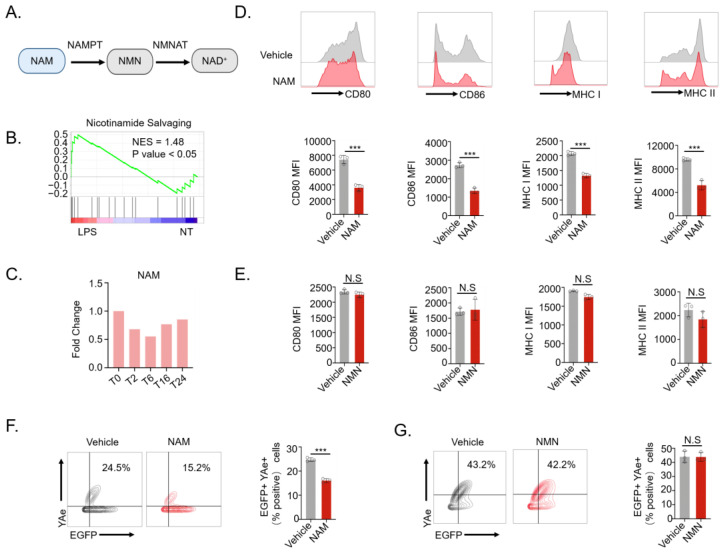
NAM inhibits DCs maturation and Ag presentation. (**A**) Schematic illustration of the NAM salvage pathway. (**B**) GSEA analysis of NAM salvage pathway in LPS-stimulated activated DCs from the GEO dataset (GSE185881). (**C**) Relative abundances of NAM in LPS-stimulated DCs at different time points. (**D**) Representative flow overlay histograms and statistical analysis of the expression levels of CD80, CD86, MHC-I, and MHC-II in vehicle- or NAM-treated BMDCs after stimulation with LPS (*n* = 3). (**E**) Statistical analysis of the expression levels of CD80, CD86, MHC-I, and MHC-II in vehicle- or NMN-treated BMDCs after stimulation with LPS (*n* = 3). (**F**) The percentage of EGFP^+^YAe^+^ cells in vehicle- or NAM-treated BMDCs after in vitro antigen uptake and presentation assay with Eα peptide (*n* = 3). (**G**) The percentage of EGFP^+^YAe^+^ cells in vehicle- or NMN-treated BMDCs after in vitro antigen uptake and presentation assay with Eα peptide (*n* = 3). Data are presented as means ± SD. *** *p* < 0.001; NS, not significant. Unpaired two-tailed Student’s *t*-test.

To explore metabolic pathways involved in DCs activation, we conducted RNA-seq analysis on LPS-activated DCs using a public dataset (GEO: GSE185881). Transcriptome profiling and Gene Set Enrichment Analysis (GSEA) revealed significant upregulation of the nicotinamide salvaging pathway, which correlated with activation of LPS-stimulated bone marrow-derived dendritic cells (BMDCs) (Figure 1A,B and Supplementary Table S4). Using RT-qPCR, we confirmed the upregulation of key enzyme NAMPT in LPS-stimulated BMDCs (Supplementary Figure S1A), suggesting a potential decrease in NAM levels during DCs activation. To further investigate the NAM level in activated DCs, we performed a metabolomics analysis based on raw data from a previous study, which utilized an untargeted metabolomics approach to explore the metabolic changes in dendritic cells upon lipopolysaccharide activation [27]. The results revealed an initial decrease in NAM levels in LPS-stimulated BMDCs (Figure 1C and Supplementary Table S5). These results encouraged us to explore the impact of NAM on DC activation. Flow cytometry and RT-qPCR analyses revealed that NAM significantly reduced the maturity of LPS-stimulated BMDCs, as indicated by the downregulation of CD80, CD86, MHC I, and MHC II expression (Figure 1D and Supplementary Figure S1B).

As known, NAM is metabolized into Nicotinamide Mononucleotide (NMN), which eventually converts to NAD^+^ (Figure 1A). To investigate whether NAM’s regulation of DC function depends on its metabolic pathway, we utilized NMN as a parallel control in an in vitro DC functional model. Interestingly, NMN did not significantly affect the maturation of DCs (Figure 1E and Supplementary Figure S1C). Furthermore, using the Eα-YAe antigen presentation model [28], we found that NAM, as opposed to NMN, significantly inhibited the antigen-presenting ability of DCs (Figure 1F,G), suggesting that NAM modulates DCs activation independent of NAD^+^. Moreover, treatment with NAM significantly decreased LPS-induced expression of pro-inflammatory cytokines (IFN-γ, IL-1β, and IL-6) and chemokine (chemokine receptor 7, CCR7) in BMDCs (Supplementary Figure S1D).

**Figure 2:**
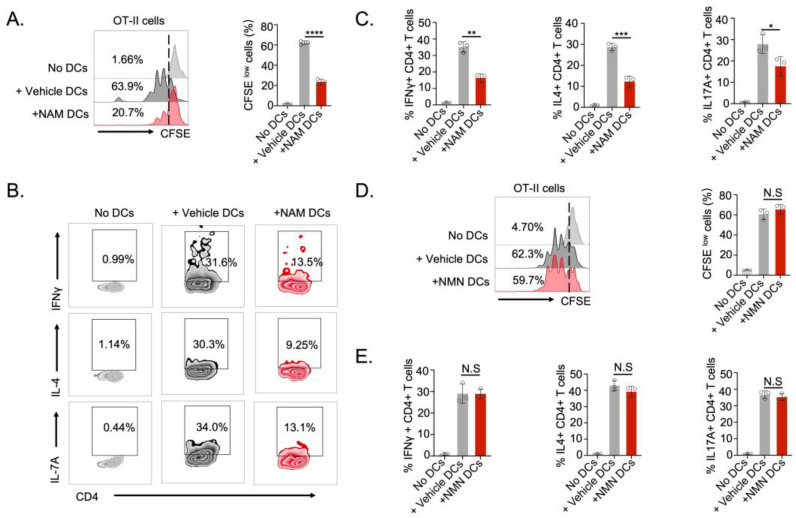
NAM impairs DC-mediated T cell priming. (**A**) Proliferation rates of CFSE-labeled OT-II cells incubated with vehicle- or NAM-treated BMDCs pulsed with OVA_323–339_ at day 3 (*n* = 3). (**B**,**C**) Representative flow staining and quantification of IFNγ^+^, IL4^+,^ or IL17A^+^ OT-II cells incubated with vehicle- or NAM-treated BMDCs pulsed with OVA_323–339_ (*n* = 3). (**D**) Proliferation rates of CFSE-labeled OT-II cells incubated with vehicle- or NMN-treated BMDCs pulsed with OVA_323–339_ at day 3 (*n* = 3). (**E**) The Flow cytometry analysis and quantification of IFNγ^+^, IL4^+,^ or IL17A^+^ OT-II cells incubated with vehicle- or NMN-treated BMDCs pulsed with OVA_323–339_ (*n* = 3). Data are presented as means ± SD. * *p* < 0.05, ** *p* < 0.01, *** *p* < 0.001, **** *p* < 0.0001; NS, not significant. Unpaired two-tailed Student’s *t*-test.

To further validate the impact of NAM on the antigen-presenting capability of DCs, we used OVA-specific CD4^+^ T cells (OT-II cells) and BMDCs co-culture model. Notably, NAM obviously inhibited DCs and triggered the proliferation of OT-II cells, as measured by CFSE staining (Figure 2A). In addition, NAM-treated DCs exhibited an impaired ability to initiate the production of IFN-γ, IL-17, and IL-4 in OT-II cells (Figure 2B,C). Consistently, DCs cultured with or without NMN showed similar capacity in priming T cell activation, as demonstrated by T cell proliferation (Figure 2D) and cytokine production (Figure 2E and Supplementary Figure S2A–C). Taken together, these results indicate a novel role of endogenous metabolite NAM in modulating DCs function beyond its metabolic conversion pathway.

### 3.2. NAM Ameliorates the Psoriasis-like Skin Inflammation through Reducing DCs Mediated Immune Response in IMQ-Induced Mouse Model

**Figure 3:**
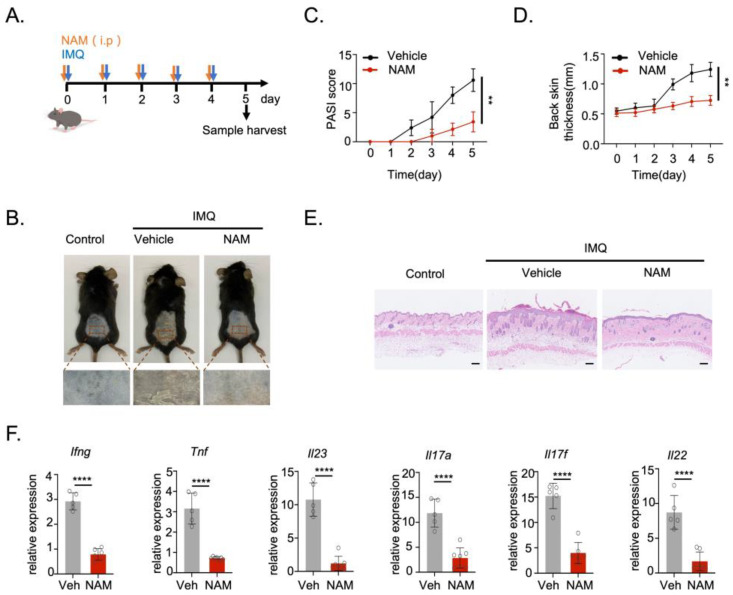
NAM ameliorates the psoriasis-like skin inflammation in IMQ-induced mouse models. (**A**) Schematic diagram of psoriasis-like mouse model induction. Mice were topically treated with 62.5 mg IMQ on shaved back skin daily for 6 consecutive days. NAM (300 mg/kg per mouse) or PBS was intravenously injected into the mice 1 h before IMQ treatment on 6 consecutive days. Samples were harvested on day 6 for subsequent experiments. (**B**) Representative photos of mouse back skin treated with vehicle or NAM (*n* = 5). (**C**) Daily clinical scores (PASI) for disease severity from IMQ-induced C57BL/6 mice treated with vehicle or NAM (*n* = 5). (**D**) Back thickness of IMQ-induced C57BL/6 mice treated with vehicle or NAM (*n* = 5). (**E**) H&E staining of skin sections of IMQ-induced C57BL/6 mice treated with vehicle or NAM (*n* = 5). Scale bar = 100 μm. (**F**) Expression of pathogenic factors in the skin of IMQ-induced C57BL/6 mice treated with vehicle or NAM as determined by quantitative PCR (*n* = 5). Data are presented as means ± SD. ** *p* < 0.01, **** *p* < 0.0001. Unpaired two-tailed Student’s *t*-test.

**Figure 4:**
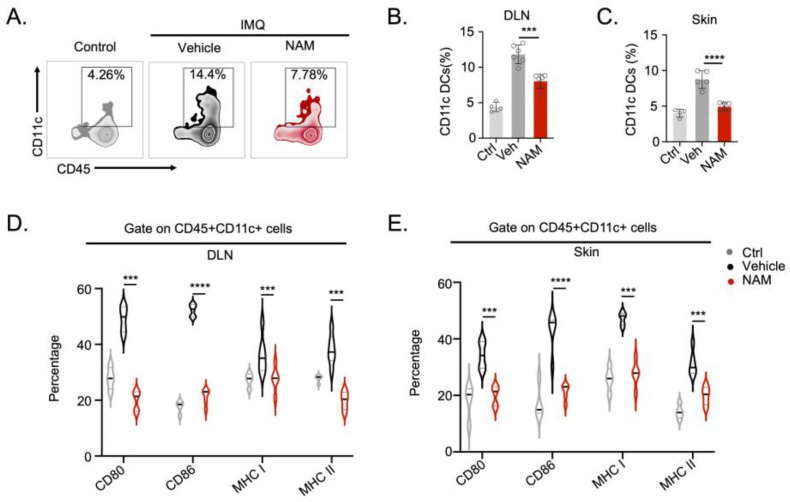
NAM inhibits DCs activation and infiltration in psoriasiform skin inflammation. (**A**,**B**) Representative flow cytometry plots (**A**) and statistical analysis (**B**) of CD45^+^CD11c^+^ cells in Draining lymph nodes (DLN) from IMQ-induced C57BL/6 mice treated with vehicle or NAM (*n* = 5). (**C**) Statistical analysis of CD45^+^CD11c^+^ cells in the skin from IMQ-induced C57BL/6 mice treated with vehicle or NAM (*n* = 5). (**D**,**E**) The statistical analysis of DC activation in DLN (**D**) and skin (**E**) on day 6 from IMQ-induced C57BL/6 mice treated with vehicle or NAM (*n* = 5). Data are presented as means ± SD. *** *p* < 0.001, **** *p* < 0.0001. Unpaired two-tailed Student’s *t*-test.

Aberrant activation of DCs plays pivotal roles in the development of various autoimmune diseases, including psoriasis. To further analyze the role of NAM in regulating autoimmunity in vivo, we employed an imiquimod (IMQ)-induced mouse model of psoriasis. 5% IMQ cream was applied daily to the back of C57BL/6 mice for six consecutive days. NAM or PBS was administered via daily intraperitoneal injection (Figure 3A). Remarkably, NAM treatment significantly alleviated the PASI score and reduced the thickness of the back skin in the IMQ mouse model (Figure 3B–E). Subsequently, we investigated whether NAM influences the expression of inflammatory cytokines in the skin. Indeed, the mRNA levels of key pathogenic factors in the skin, including Ifng, Tnf, Il23, Il17a, Il17f, and Il22, were significantly reduced after NAM treatment (Figure 3F). In addition, NAM reversed the spleen swelling in the IMQ mouse model (Supplementary Figure S3A,B). Importantly, the percentage of CD45^+^ CD11c^+^ DCs was markedly decreased in the draining lymph node (DLN) and skin of mice exposed to NAM management (Figure 4A–C and Supplementary Figure S4). Moreover, the activity of migrated DCs, as indicated by expression of CD80, CD86, MHC I, and MHC II on the cell surface, was also decreased in the dermis and DLNs of NAM-treated mice, as compared to the control group (Figure 4D,E). Collectively, these findings indicate that NAM limits DCs activation and alleviates psoriasis-like skin inflammation in vivo.

### 3.3. NAM Suppresses DCs Activation through PARP Dependent NF-κB Signaling Pathway

**Figure 5:**
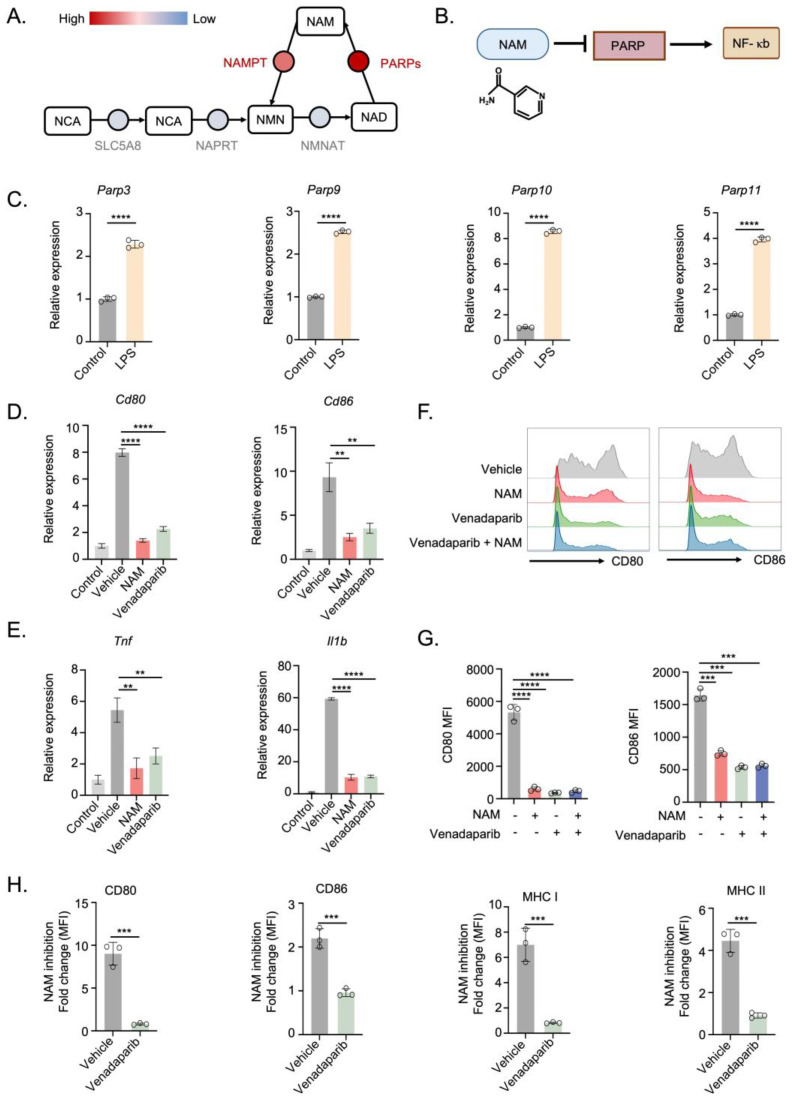
NAM regulates DC activation through a PARP-dependent NF-κB signaling pathway. (**A**) RNA expression levels of NAM salvage pathway-related genes in LPS-stimulated activated DCs (GSE235310). (**B**) Schematic illustration of the regulation between NAM, PARP, and NF-κB. (**C**) mRNA expression of Parp3, Parp9, Parp10 and Parp11 in vehicle- or LPS-stimulated BMDCs (*n* = 3). (**D**) mRNA expression of Cd80 and Cd86 in vehicle-, NAM- or PARP inhibitor (Venadaparib)-treated BMDCs after stimulation with LPS (*n* = 3). (Control: without LPS stimulation). (**E**) mRNA expression of Tnf and Il1b in vehicle-, NAM- or Venadaparib-treated BMDCs after stimulation with LPS (*n* = 3). (Control: without LPS stimulation). (**F**,**G**) Representative flow overlay histograms (**F**) and statistical analysis (**G**) of BMDCs treated as indicated and stimulated by LPS (*n* = 3). (**H**) Fold change for the inhibitory ability of NAM in vehicle- or Venadaparib-treated BMDCs (*n* = 3). Data are presented as means ± SD. ** *p* < 0.01, *** *p* < 0.001, **** *p* < 0.0001. Unpaired two-tailed Student’s *t*-test.

To explore the mechanisms by which NAM impairs TLR-triggered DC activation, we analyzed the gene expression patterns in LPS-activated DCs using the published sequencing data. In the dataset deposited in GSE235310, we found the expression of PARPs in the NAM salvage pathway was significantly upregulated during LPS-stimulated DCs activation (Figure 5A). Due to the critical regulation of PARPs on NF-κB activity [29], upregulation of PARPs could enhance NF-κB activation, thereby contributing to DCs activation. Given the known role of NAM as a PARP inhibitor [30], we propose a hypothesis that NAM impairs DCs activation by inhibiting PARPs, thereby downregulating the critical NF-κB signaling pathway (Figure 5B). Before testing this possibility, we first validated the upregulation of PARP family genes in LPS-stimulated BMDCs through qRT-PCR analysis. Our results demonstrated that a series of PARP genes were significantly elevated in LPS-stimulated BMDCs (Figure 5C and Supplementary Figure S5A). To examine the role of PAPRs in DCs regulation, we employed the well-established PARP inhibitor Venadaparib. Similar to NAM treatment, Venadaparib significantly reduced the expression of DC marker genes (such as CD80 and CD86) in LPS-stimulated BMDCs. Additionally, Venadaparib notably inhibited the expression of NF-κB-related genes (including TNF, IL-1, IL-6 and CCL2), which are crucial for DCs activation (Figure 5D,E and Supplementary Figure S5B). These results suggest that PARP activation is essential for DC function.

Building on these results, we further examined whether NAM’s effects on DCs activation were dependent on PARP activity. BMDCs were pretreated with NAM alone or with a combination of Venadaparib and NAM, and their activation status was assessed. Flow cytometry analysis revealed that NAM treatment significantly suppressed DCs activation. However, in DCs pre-incubated with the PARP inhibitor, NAM did not exert additional suppression of DC function (Figure 5F–H and Supplementary Figure S5C,D), indicating that NAM’s impairment of DC activation depends on PARP activity.

### 3.4. NAMPT Is Activated in DCs of Psoriasis and the NAMPT-PARP Axis Predicts Higher PASI Score

**Figure 6:**
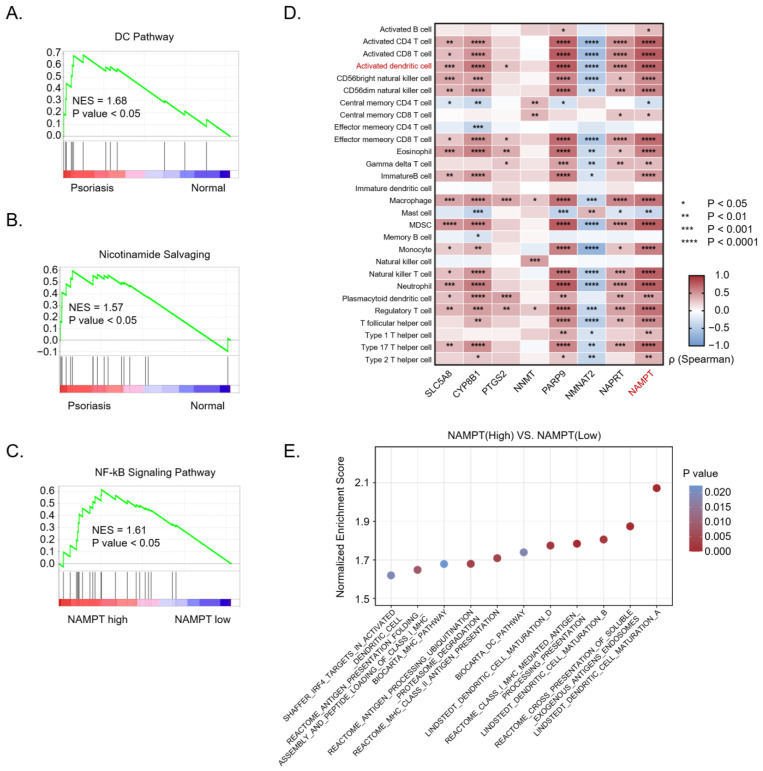
NAMPT is obviously upregulated during DCs activation in psoriasis. (**A**) GSEA analysis of DCs pathway in skin samples from psoriasis patients and healthy controls from the GEO dataset (GSE121212). (**B**) GSEA analysis of NAM salvage pathway in skin samples from psoriasis patients and healthy controls from the GEO dataset (GSE121212). (**C**) GSEA analysis of NF-κB signaling pathway between NAMPT high- and low-level groups in clinical samples from the GEO dataset (GSE121212). (**D**) Correlation heatmap of NAM salvage pathway-related genes with the infiltration levels of various immune cell types in the skin of psoriasis patients and healthy controls from the GEO dataset (GSE121212). (**E**) GSEA analysis of DCs function-related gene sets in skin samples from psoriasis patients and healthy controls from the GEO dataset (GSE121212).

To further explore the relationship between DCs activation and the NAMPT-PARP axis in clinical samples, we reanalyzed a publicly accessible transcriptomic dataset of skin tissues from psoriasis patients (GEO: GSE121212). GSEA analysis demonstrated a significant correlation between DCs activation pathways and psoriasis, supporting that DCs are hyperactivated in psoriasis patients (Figure 6A and Supplementary Figure S6). Furthermore, as in activated DCs (Figure 1B), we similarly observed the upregulation of nicotinamide salvaging pathway in psoriasis patient samples (Figure 6B), suggesting lower NAM levels in psoriasis patients, consistent with previous findings that levels of NAM, or its precursor metabolite nicotinic acid, are decreased in patients with RA, SLE, and psoriasis [31,32,33]. Additionally, we found the patient group with higher-level NAMPT demonstrated stronger activation of the NF-κB pathway (Figure 6C). These results prompt us to explore whether NAMPT expression is correlated with the DCs hyperactivation in psoriasis patients. To investigate this possibility, we analyzed the correlation between NAM metabolism-related enzyme expression and immune infiltration of activated DCs in psoriasis patients. Interestingly, a significant positive correlation between NAMPT expression and the immune infiltration of activated DCs was observed (Figure 6D). Using the human gene list for DCs maturation/activation from the Molecular Signature Database, we found that NAMPT showed a strong correlation with the activated DCs signature through GSEA analysis (Figure 6E). Thus, NAMPT higher expression appears closely linked to DCs’ overactivation in autoimmune disease pathology.

**Figure 7:**
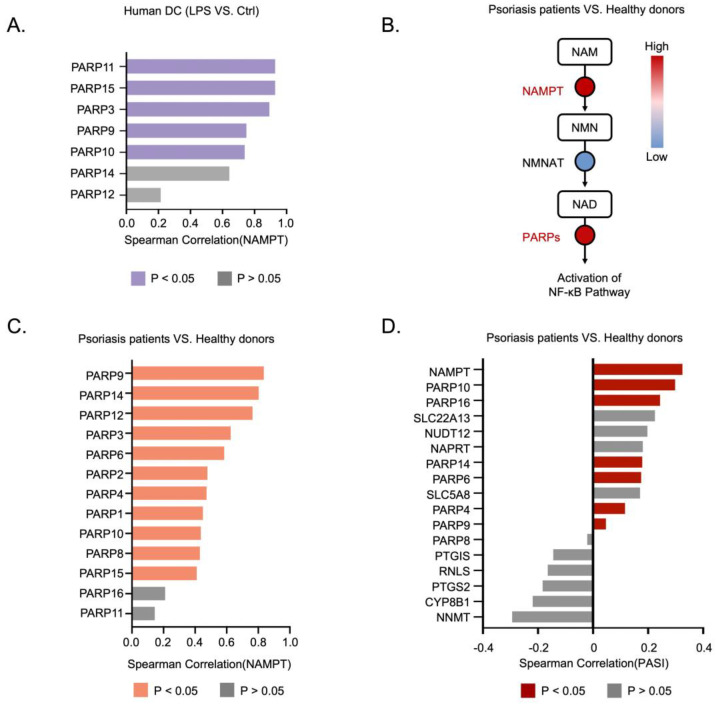
Upregulation of the NAMPT-PARP axis in DCs and psoriasis predicts higher PASI score. (**A**) Correlation of RNA expression levels between NAMPT and PARP family genes in LPS-stimulated DCs and control cells from the GEO dataset (GSE235310). (**B**) RNA expression levels of NAM salvage pathway-related genes in skin samples from psoriasis patients and healthy controls from the GEO dataset (GSE121212). (**C**) Correlation of RNA expression levels between NAMPT and PARP family genes in skin samples from psoriasis patients and healthy controls from the GEO dataset (GSE121212). (**D**) Correlation between PASI score and RNA expression levels of NAM salvage pathway-related genes from the GEO dataset (GSE121212).

Based on the dataset, we observed that NAMPT and PARP family genes were significantly upregulated in LPS-stimulated DCs. Next, we examined the potential connection between NAMPT and PARPs. Through reanalyzing the RNA-seq dataset of LPS-stimulated human DCs, we found a robust positive correlation between the expression of NAMPT and PARPs (Figure 7A). Consistently, in publicly available clinical datasets of psoriasis, we also observed significant upregulation of NAMPT and PARP genes in skin samples, with a notable correlation between their expressions (Figure 7B,C). GSEA analysis confirmed the positive association of PARPs expression with the NF-κB signaling pathway in psoriasis patients (Supplementary Figure S7A,B). Since NAM impairs DCs function by inhibiting PARPs activation and considering the significant correlation between NAMPT and PARPs, we examined whether the NAMPT-PARP axis correlated with the PASI score in psoriasis patients. Indeed, our analysis revealed that higher PASI scores were presented in patients with elevated expression levels of NAMPT and PARP family genes (Figure 7D). Collectively, these findings indicate that NAMPT could be a promising target for suppressing activated DCs in psoriasis and that the NAMPT-PARP axis serves as a predictive biomarker for severe psoriasis.

## 4. Discussion

DCs are pivotal in bridging innate immunity with adaptive immune responses. Therapeutics targeting DCs represent a vibrant and promising field in immunology, aiming to modulate DC function for enhancing immune responses against infections and cancer or mitigating autoimmune diseases [3,34]. Previous studies on DCs have predominantly targeted genes, proteins, or cytokine inhibitors as key therapeutic avenues [35,36,37]. However, recent findings highlight the critical role of endogenous metabolites in modulating DCs function [38]. Alterations in metabolic pathways, along with variations in metabolites and nutrients, have been noted across different DCs subsets and functions [39]. Given the pivotal influence of metabolic reprogramming on DCs function, there is a growing interest in metabolic molecules for the regulation of DCs. The higher safety profile of natural metabolites makes them a potentially advantageous therapeutic target for DCs modulation. While some metabolites have been identified to regulate DCs function in tumor therapy [4], their regulatory effects on DCs in the context of autoimmune diseases warrant further investigation.

NAM, a derivative of vitamin B3, can be converted into NAD^+^ through salvage pathways in cells, where it plays a crucial role in maintaining cellular redox balance and metabolic homeostasis. The coenzyme NAD^+^ is crucial for energy production, DNA repair, and cellular metabolism. Current research on nicotinamide primarily focuses on its role as a precursor of NAD^+^ in regulating anti-tumor immunity and protecting against organ damage [13,14]. However, research directly investigating the role of NAM in immune regulation remains limited. Previous studies have suggested NAM’s potential anti-inflammatory and antioxidant effects, as well as its possible application in treating various inflammatory diseases [15,40]. Nevertheless, the efficacy of NAM specifically in treating psoriasis has not been thoroughly studied, and the precise mechanisms through which NAM operates in this therapeutic context remain unexplored. In our current research, we demonstrated the crucial role of NAM in regulating DC activation. Importantly, the inhibition of DCs is mediated specifically by NAM itself rather than its product NAD^+^, as NMN did not affect DCs function. Our in vivo experiments demonstrated that NAM can effectively mitigate IMQ-induced psoriasis in mice by inhibiting DCs function. Notably, clinical data analysis consistently revealed a significant correlation between NAMPT expression and DCs infiltration in psoriasis patient samples.

Since NAD^+^ can be metabolized into NAM through the action of PARP enzymes, making NAM a byproduct of PARP activity. In our study, bioinformatics analysis of sequencing data revealed significant upregulation of NAMPT and PARPs in activated DCs and psoriasis patient samples. Given the critical roles of NAMPT in NAM consumption and PARPs in NAM accumulation, we assessed the actual levels of NAM during DC activation. Our analysis of metabolomics data indicates that NAM is significantly downregulated in activated DCs and in patients with autoimmune diseases. This reduction in NAM may reflect a balance resulting from the upregulation of these two genes.

In this study, we found that NAM inhibits the PARP-mediated activation of the NF-κB signaling pathway, thereby restraining DCs function. This suggests that NAM may also regulate other immune cells activated through the NF-κB signaling pathway, warranting further research for validation in the future. Moreover, exploring whether NAM can serve as a candidate drug for treating other DC-related autoimmune diseases, such as rheumatoid arthritis, is promising. Future work could expand the scope of research on NAM in the field of immunology to enhance its therapeutic value in autoimmune diseases. Based on the analysis of publicly available clinical databases, we observed a close link between the NAMPT-PARP axis and psoriasis, which provides a new predictor for PASI score in psoriasis. Clinically, evaluating the expression levels of the NAMPT-PARP axis can potentially characterize disease severity.

## 5. Conclusions

This study identified NAM as a natural suppressor of DC activation, showing that it limits NF-κB-mediated gene expression through a PARP-dependent mechanism. This suppression effectively restrains DC maturation and impairs T cell activation both in vitro and in vivo. These findings not only elucidate the specific therapeutic mechanism of NAM in treating psoriasis but also highlight the NAMPT-PARP axis as a promising predictive biomarker for autoimmune diseases. Future studies could explore whether NAM affects other types of immune cells and if its suppressive effects on DC activation can be leveraged to treat a broader range of autoimmune and inflammatory conditions.

## Data Availability

The expression matrix of psoriasis patients and healthy donors is available in the NCBI Gene Expression Omnibus (GEO) database (GSE121212). The expression matrix of mouse BMDCs stimulated with or without LPS is available in the GEO database (GSE185881). The expression matrix of human monocyte-derived dendritic cells (moDCs) stimulated with or without LPS is available in the GEO database (GSE235310). All additional data supporting this study can be found within this article, the Supplementary Information, the source data, or can be obtained from the corresponding authors upon reasonable request. Source data are provided in this paper.

## Supplementary Materials

The following supporting information can be downloaded at: https://www.mdpi.com/10.3390/nu16162665/s1, Figure S1: NAM inhibits DCs maturation and Ag presentation; Figure S2: NAM impairs DC-mediated T cell priming; Figure S3: NAM ameliorates the psoriasis-like skin inflammation in IMQ-induced mouse model; Figure S4: NAM inhibits DCs activation and infiltration in psoriasiform skin inflammation; Figure S5: NAM regulates DCs activation through PARP dependent NF-κB signaling pathway; Figure S6: NAMPT is obviously upregulated during DCs activation in psoriasis; Figure S7: Upregulation of the NAMPT-PARP axis in DCs and psoriasis predicts higher PASI score; Table S1: Antibodies used for Cell detection in flow cytometry; Table S2: Polyclonal antibodies used in this study; Table S3: Primers for real-time polymerase chain reaction; Table S4: Transcriptomics Alterations in the Nicotinamide Salvaging Pathway; Table S5: Metabolomics Analysis for NAM and NADH.
