# Supplementary material for: Nicotinamide Suppresses Hyperactivation of Dendritic Cells to Control Autoimmune Disease through PARP Dependent Signaling
Source: Nutrients. 2024 Aug 12;16(16):2665. doi: 10.3390/nu16162665 (PMC11356829; doi:10.3390/nu16162665)
Supplement: Supplementary file 1 [file nutrients-16-02665-s001.zip › Supplementary Information.pdf]

Nicotinamide suppresses hyperactivation of dendritic cell to control autoimmune disease through PARP dependent signaling

Supplemental Figure legends

Supplementary Figure 1. NAM inhibits DCs maturation and Ag presentation

A. mRNA expression of *Nampt* in vehicle- or LPS-stimulated BMDCs (n = 3).

B. mRNA expression of *Cd80* and *Cd86* in vehicle- or NAM-treated BMDCs after stimulation with LPS (n=3).

C. Representative flow overlay histograms of CD80, CD86, MHC-I and MHC-II in vehicle- or NMN-treated BMDCs after stimulation with LPS (n=3).

D. mRNA expression of *Ifng*, *Il1b*, *Il6* and *Ccr7* in vehicle- or NAM-treated BMDCs after stimulation with LPS (n=3). Data are presented as means ± SD. *P < 0.05, **P < 0.01, ***P < 0.001, ****P < 0.0001; NS, not significant. Unpaired two-tailed Student's t-test.

Supplementary Figure 2. NAM impairs DC-mediated T cell priming

A-C. Representative flow staining of IFNγ^+ (a), IL4$^+$ (b) or IL17A$^+$ (c) OT-II cells incubated with vehicle- or NMN-treated BMDCs pulsed with $OVA_{323-339}$ (n=3). Data are presented as means ± SD. *P < 0.05, **P < 0.01, ***P < 0.001, ****P < 0.0001; NS, not significant. Unpaired two-tailed Student's t-test.

Supplementary Figure 3. NAM ameliorates the psoriasis-like skin inflammation in IMQ-induced mouse model

A-B. The photos (a) and weight (b) of spleen from IMQ-induced C57BL/6 mice treated with vehicle or NAM (n = 5). Data are presented as means ± SD. *P < 0.05, **P < 0.01, ***P < 0.001, ****P < 0.0001; NS, not significant. Unpaired two-tailed Student's t-test.

Supplementary Figure 4. NAM inhibits DCs activation and infiltration in psoriasiform skin inflammation

Representative flow cytometry plots of $CD45^{+}CD11c^{+}$ cells in skin from IMQ-induced C57BL/6 mice treated with vehicle or NAM (n = 5). Data are presented as means ± SD. *P < 0.05, **P < 0.01, ***P < 0.001, ****P < 0.0001; NS, not significant. Unpaired two-tailed Student's t-test.

Supplementary Figure 5. NAM regulates DCs activation through PARP dependent NF-κB signaling pathway

A. mRNA expression of *Parp12* and *Parp14* in vehicle- or LPS-stimulated BMDCs (n = 3).

B. mRNA expression of *Il12a, Il6, Nfkbia, Ccl5*, *Ccl2* and *Il23* in vehicle-, NAM- or PARP inhibitor (Venadaparib)-treated BMDCs after stimulation with LPS (n=3). (Control: without LPS stimulation)

C-D. Representative flow overlay histograms (c) and statistical analysis (d) of BMDCs treated as indicated and stimulated by LPS (n=3). Data are presented as means ± SD. *P < 0.05, **P < 0.01, ***P < 0.001, ****P < 0.0001; NS, not significant. Unpaired two-tailed Student's t-test.

Supplementary Figure 6. NAMPT is obviously upregulated during DCs activation in psoriasis

Infiltration levels of various immune cell types in the skin of psoriasis patients and healthy controls (GSE121212).

Supplementary Figure 7. Upregulation of NAMPT-PARP axis in DCs and psoriasis predicts higher PASI score

A. GSEA analysis of NF-κB signaling pathway between PARP9 high- and PARP9 low-level groups in clinical samples from the GEO dataset (GSE121212).

B. GSEA analysis of NF-κB signaling pathway between PARP12 high- and PARP12 low- level groups in clinical samples from the GEO dataset (GSE121212).

Supplementary Tables

Table S1. Antibodies used for Cell detection in flow cytometry

Marker antibody	Cells	Objective
Biotin-conjugated anti-Eα 52–68 peptide	DCs	Antigen presentation
APC Streptavidin	DCs	Antigen presentation
APC-anti-CD11c	DCs	Phenotype
PE-anti-MHC II	DCs	Maturation
FITC-anti-MHC I	DCs	Maturation
PE-anti-CD80	DCs	Maturation
FITC-anti-CD86	DCs	Maturation
Pacific Blue-anti-CD45	Leukocytes	Phenotype
PE-CD4	CD4 T cells	Phenotype
APC-IFNγ	CD4 T cells	Activation
PE-Cy7-IL17A	CD4 T cells	Activation
APC-IL4	CD4 T cells	Activation

Table S2. Polyclonal antibodies used in this study

Antibodies	Source	Identifier
APC-anti-CD11c (clone N418)	Biolegend	Cat#117310; RRID: AB_313779
PE-anti-MHC II (clone M5/114.15.2)	eBioscience	Cat#11-5321-81; RRID: AB_465232
FITC-anti-MHC I (clone 28-8-6)	Biolegend	Cat#114606; RRID: AB_313597
PE-anti-CD80 (clone 16-10A1)	Biolegend	Cat#104708; RRID: AB_313129
FITC-anti-CD86 (clone GL-1)	Biolegend	Cat#105006; RRID: AB_313149
Pacific Blue-anti-CD45 (clone 30-F11)	Biolegend	Cat#103126; RRID: AB_493535
PE-CD4 (clone GK1.5)	Biolegend	Cat#100408; RRID: AB_312693
APC-IFNγ (clone XMG1.2)	eBioscience	Cat#17-7311-82; RRID: AB_469504
PE-Cy7-IL17A (clone TC11-18H10.1)	Biolegend	Cat#506922; RRID: AB_2125010
APC-IL4 (clone 11B11)	Biolegend	Cat#17-7041-82; RRID: AB_469494

Table S3. Primers for real-time polymerase chain reaction

Mouse-gene	Forward	Reverse
Il1β	5’ GCAACTGTTCCTGAACTCAACT3’	5’ ATCTTTTGGGGTCCGTCAACT3’
Il6	5’TAGTCCTTCCTACCCCAATTTCC3’	5’ TTGGTCCTTAGCCACTCCTTC3’
Nfkbia	5’ TGAAGGACGAGGAGTACGAGC 3’	5’ TTCGTGGATGATTGCCAAGTG 3’
Ifng	5’ ATGAACGCTACACACTGCATC3’	5’ CCATCCTTTTGCCAGTTCCTC3’
Ccr7	5’ TGTACGAGTCGGTGTGCTTC3’	5’ GGTAGGTATCCGTCATGGTCTTG3’
Cd80	5’ AATCCTCCTGCACCAGTATC3’	5’ CTCTTGGAACCTCAGTGTCTTC3’
Cd86	5’ TCACCCGATACCTAAGAAGATG3’	5’ AGAGAGAGGCTGTTGGAGATA3’
Ccl5	5’GCTGCTTTGCCTACCTCTCC3’	5’TCGAGTGACAAACACGACTGC3’
Il23	5’ ATGCTGGATTGCAGAGCAGTA3’	5’ ACGGGGCACATTATTTTTAGTCT3’
Tnf	5’ GACGTGGAACTGGCAGAAGAG3’	5’ TTGGTGGTTTGTGAGTGTGAG3’
Ccl2	5’ TTAAAAACCTGGATCGGAACCAA3’	5’ GCATTAGCTTCAGATTTACGGGT3’
Il12a	5’ CTGTGCCTTGGTAGCATCTATG3’	5’ GCAGAGTCTCGCCATTATGATTC3’
Nampt	5’ GCAGAAGCCGAGTTCAACATC3’	5’ TTTTCACGGCATTCAAAGTAGGA3’
Parp3	5’ ATGGCTCCAAAACGAAAGGC3’	5’ TCCTCCTCTGTCCCTTGTCG3’
Parp9	5’ AGGACGCCAAAGGGATCTG3’	5’ CCGGCTCCATAAACTGGGT3’
Parp10	5’ CTCTGTACTTTGAAAACCACCGT3’	5’ GCTCAGTCGGACACCATGTA3’
Parp11	5’ GTGGACGACATGGACACATCG3’	5’ CCAGTGACAAGATTCATCTGCTT3’
Parp12	5’ TCATCTACGGCAACTGCAAGT3’	5’ AGCTCAGTATATGTGAGGTGGTC3’
Parp14	5’ ATTCAAGTTGACTGTCCTGATGC3’	5’ CCACATACCTTGGAATGCCAC3’
Actb	5’GGCTGTATTCCCCTCCATCG3’	5’CCAGTTGGTAACAATGCCATGT3’
Gapdh	5’ GTGTTCCTACCCCCAATGTG3’	5’ GGTCCTCAGTGTAGCCCAAG3’

Table S4. Transcriptomics Alterations in the Nicotinamide Salvaging Pathway

GENE	GSE121212		GSE235310	
	log_2(FoldChange)	P_value	log_2(FoldChange)	P_value
PTGS2	0.47	3.76E-03	0.95	3.91E-02
PARP4	0.42	4.34E-11	-0.69	5.60E-05
NAMPT	2.59	8.08E-85	2.14	2.55E-22
NUDT12	N/A	N/A	-0.69	7.06E-03
PTGIS	-2.05	6.55E-27	N/A	N/A
PARP6	0.23	6.66E-04	N/A	N/A
PARP9	2.49	3.32E-45	3.43	7.41E-90
PARP16	N/A	N/A	-2.10	3.40E-13
NAPRT	0.72	8.83E-14	N/A	N/A
PARP8	0.31	4.88E-08	-1.02	2.11E-11
NAXE	N/A	N/A	N/A	N/A
NNMT	N/A	N/A	N/A	N/A
SLC22A13	N/A	N/A	N/A	N/A
PARP14	1.79	1.05E-35	2.67	7.34E-42
PARP10	0.28	3.04E-03	2.92	1.46E-31
CYP8B1	2.01	1.83E-13	N/A	N/A
RNLS	-0.14	4.29E-02	N/A	N/A
NAXD	N/A	N/A	N/A	N/A
SLC5A8	2.15	1.03E-05	N/A	N/A

Table S5. Metabolomics Analysis for NAM and NADH

		NAM	NADH
	P_value	6.08E-01	5.92E-06
T2/T0	P_value_BH	7.44E-01	2.00E-04
	Ratio	0.68	4.17
	P_value	1.00E-02	9.77E-01
T6/T2	P_value_BH	3.45E-02	9.83E-01
	Ratio	0.81	1.03
	P_value	1.01E-01	1.01E-01
T16/T6	P_value_BH	6.66E-01	6.66E-01
	Ratio	1.39	0.51
	P_value	7.55E-01	4.43E-01
T24/T16	P_value_BH	1.00E+00	1.00E+00
	Ratio	1.11	1.20
