# Supplementary figures and images for: Nicotinamide Suppresses Hyperactivation of Dendritic Cells to Control Autoimmune Disease through PARP Dependent Signaling
Source: Nutrients. 2024 Aug 12;16(16):2665. doi: 10.3390/nu16162665 (PMC11356829; doi:10.3390/nu16162665)

A.

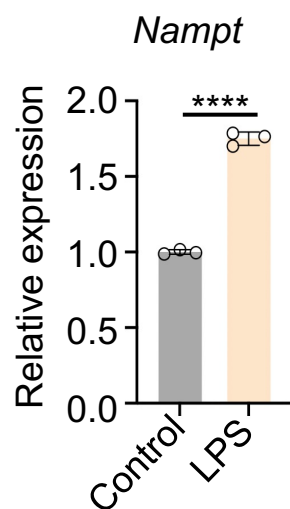

B.

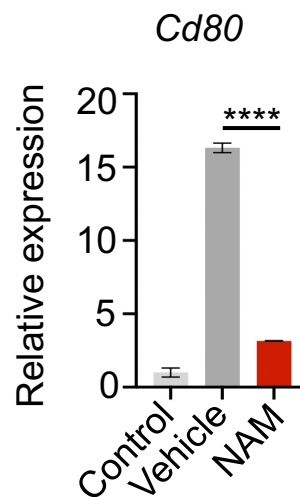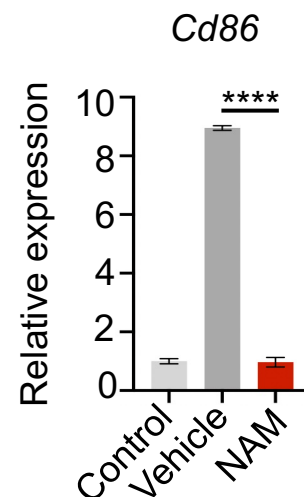

C.

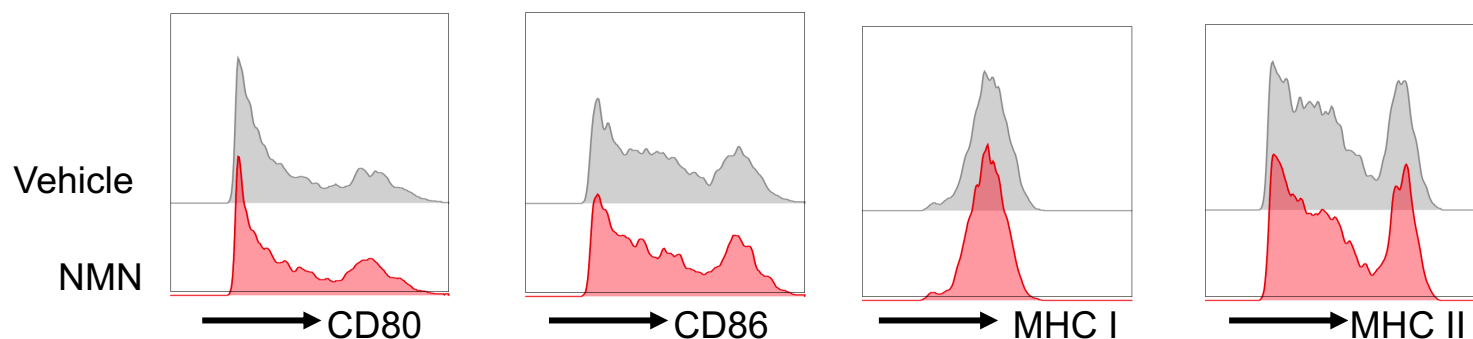

D.

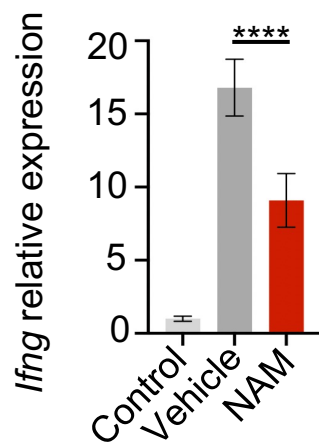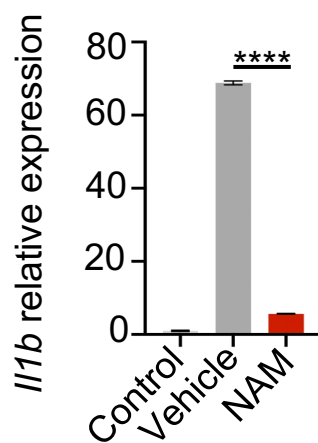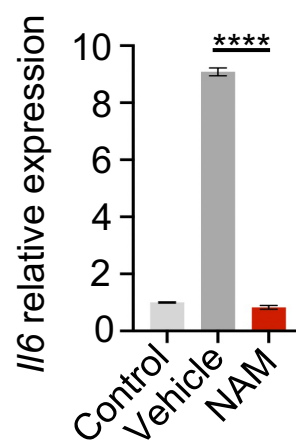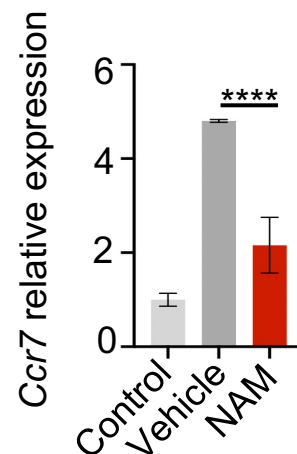

Supplement: Supplementary file 1 [file nutrients-16-02665-s001.zip › Figure S1.pdf]

A.

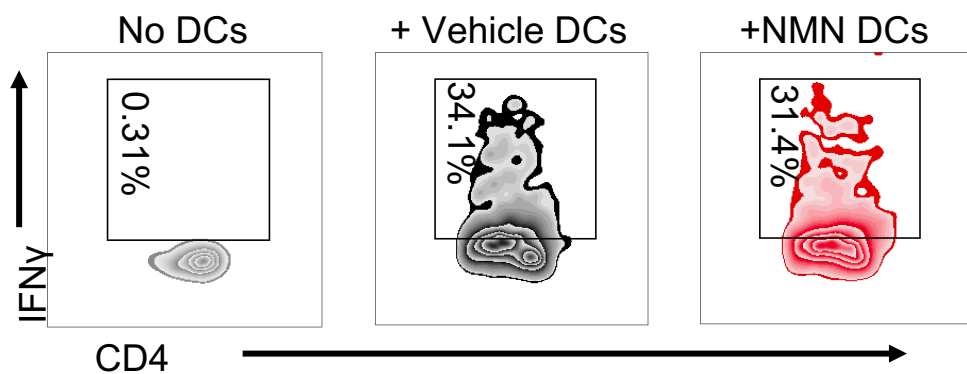

B.

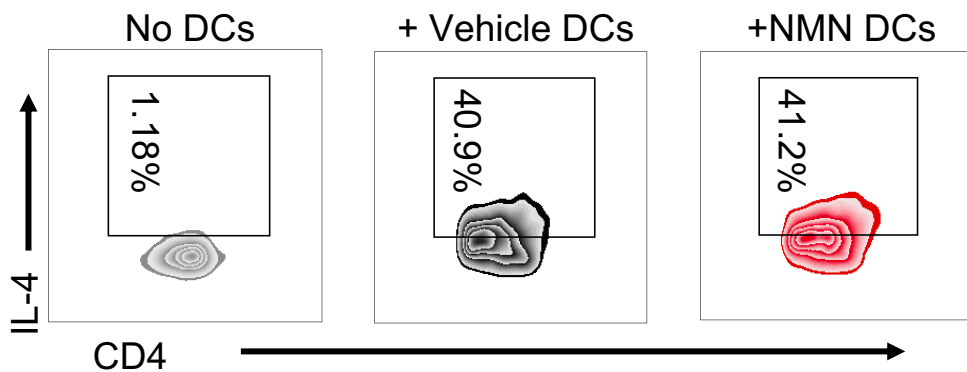

C.

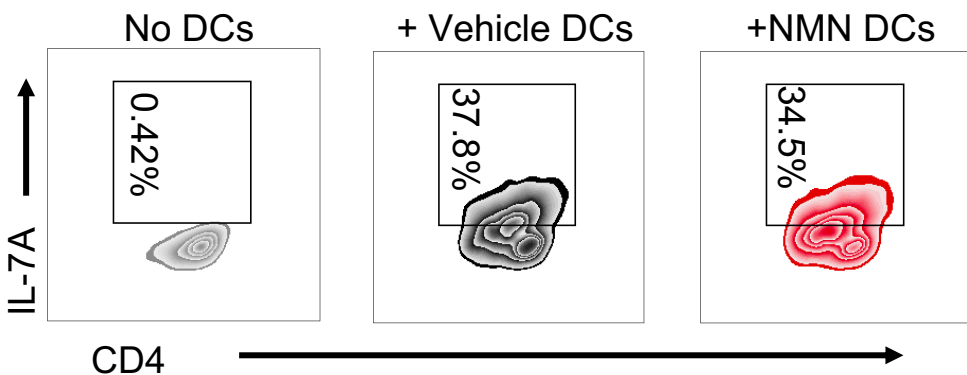

Supplement: Supplementary file 1 [file nutrients-16-02665-s001.zip › Figure S2.pdf]

A.

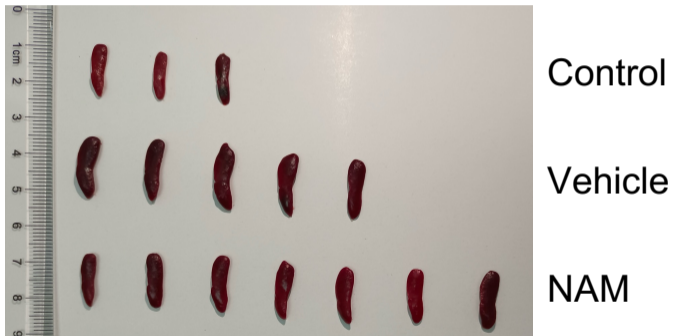

B

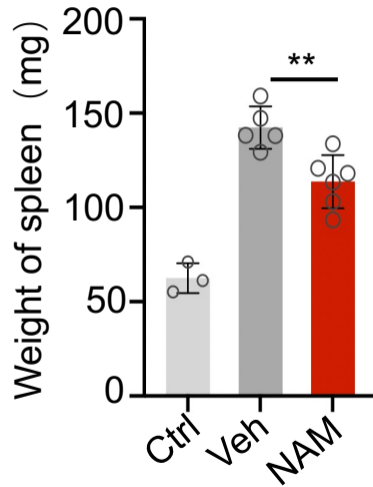

Supplement: Supplementary file 1 [file nutrients-16-02665-s001.zip › Figure S3.pdf]

CD11c

Sham

3.21%

Vehicle

9.96%

NAM

4.43%

IMQ

CD45

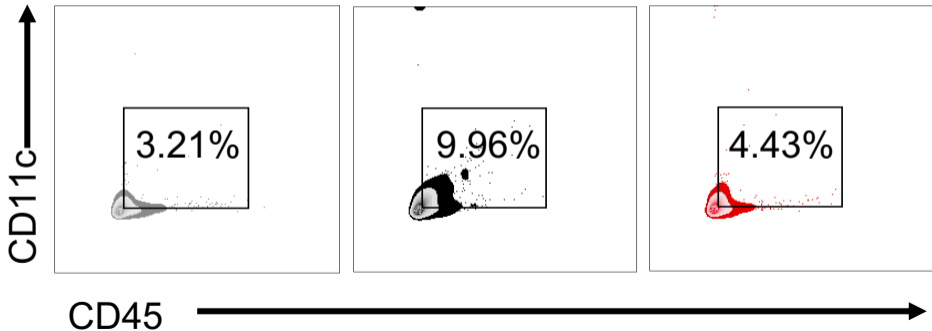

Supplement: Supplementary file 1 [file nutrients-16-02665-s001.zip › Figure S4.pdf]

A.

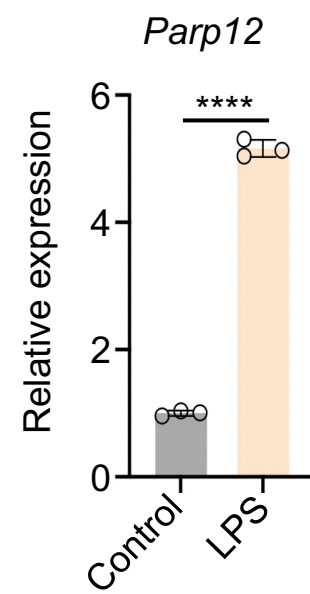

B.

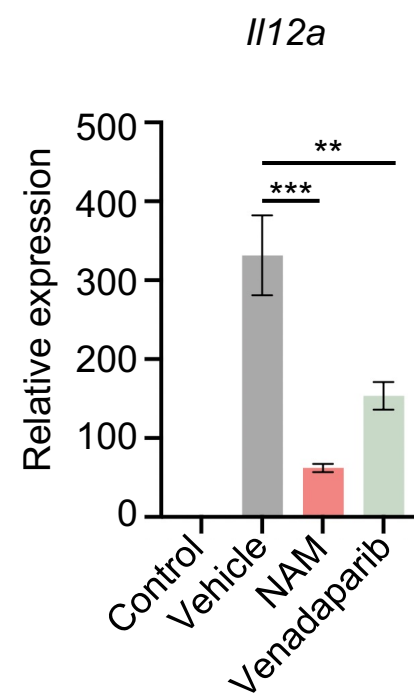*Il6*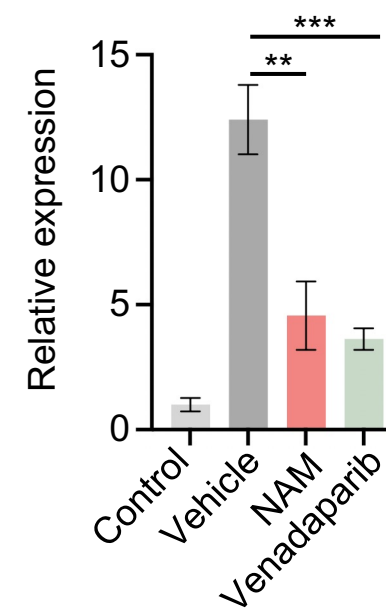*Nfkbia*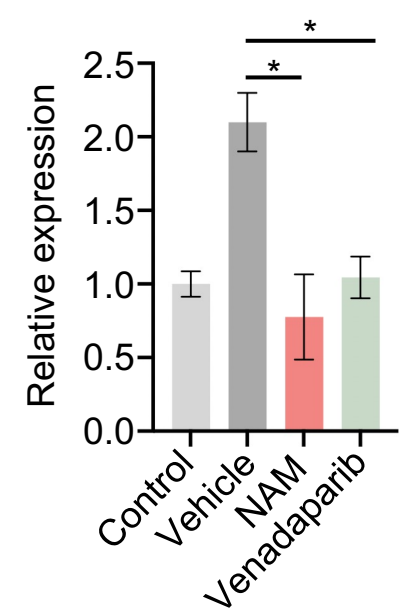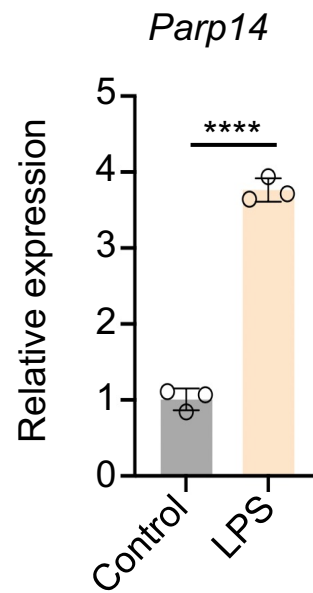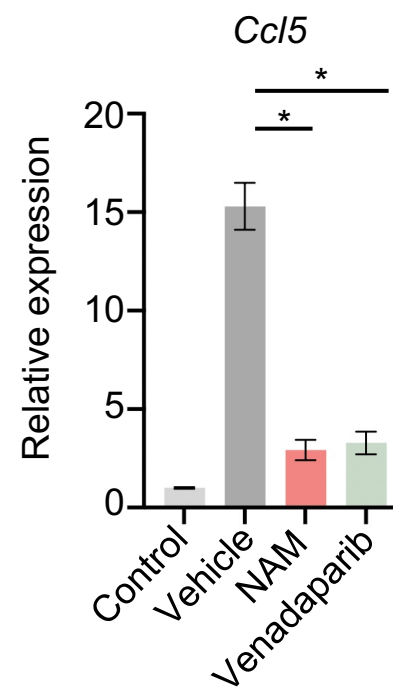*Ccl2*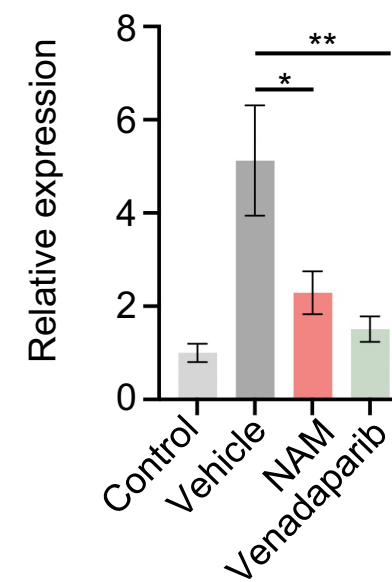*Il23*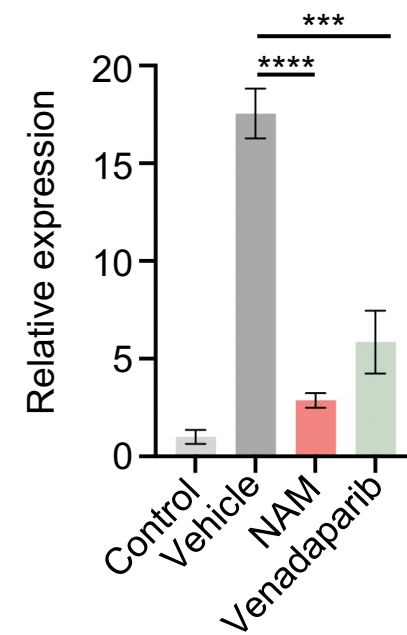

C.

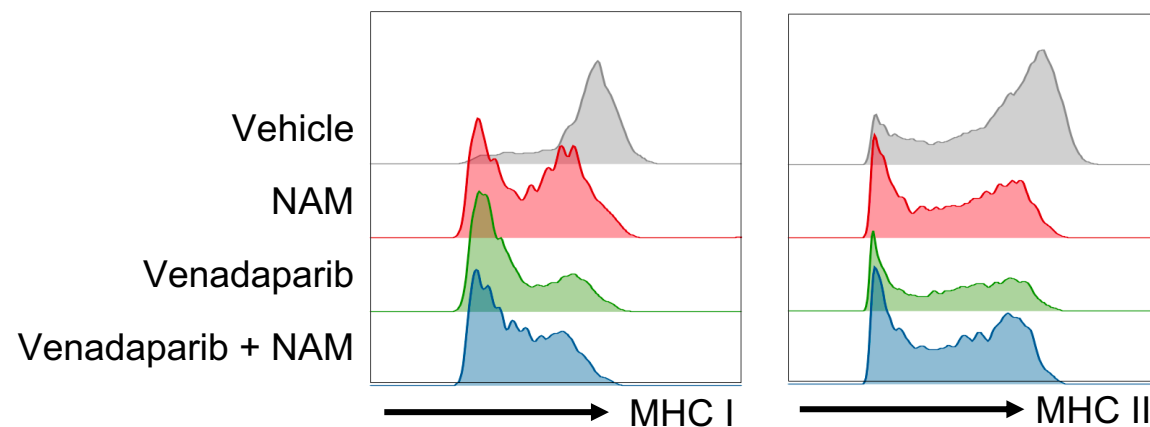

D.

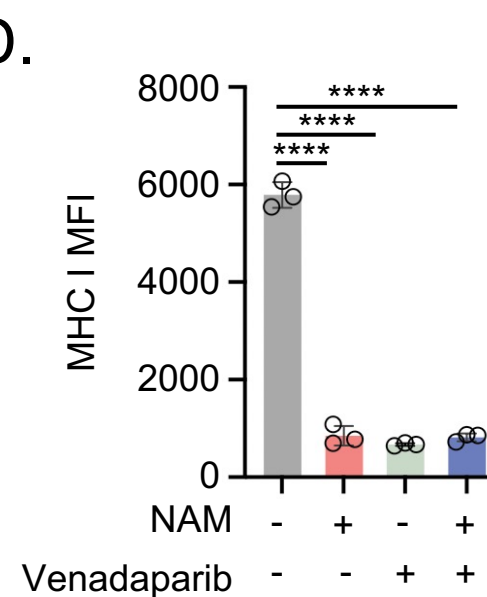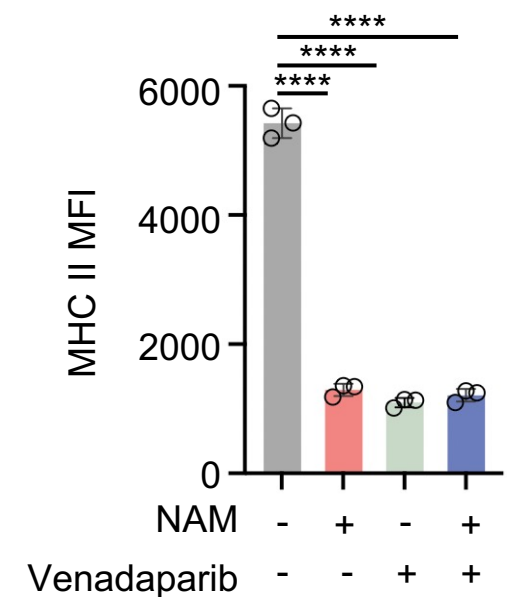

Supplement: Supplementary file 1 [file nutrients-16-02665-s001.zip › Figure S5.pdf]

Normal skin

Psoriasis skin

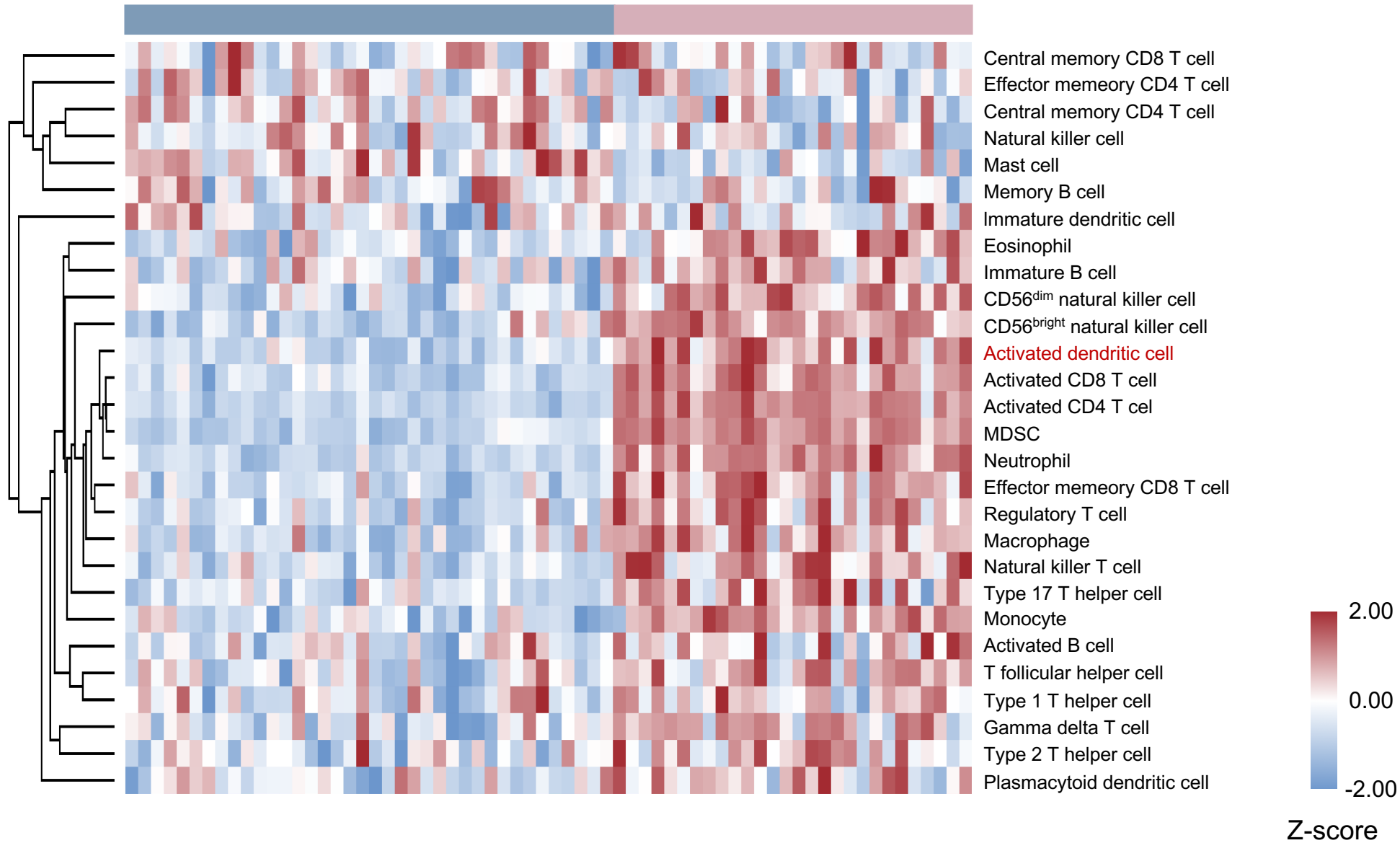

Supplement: Supplementary file 1 [file nutrients-16-02665-s001.zip › Figure S6.pdf]

# NF- $\kappa$ B Signaling Pathway

A.

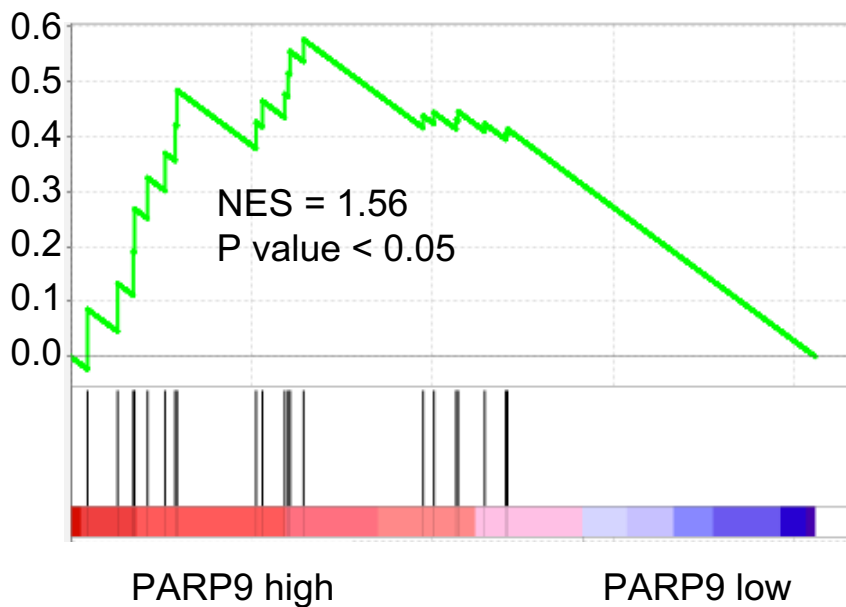

# NF- $\kappa$ B Signaling Pathway

B.

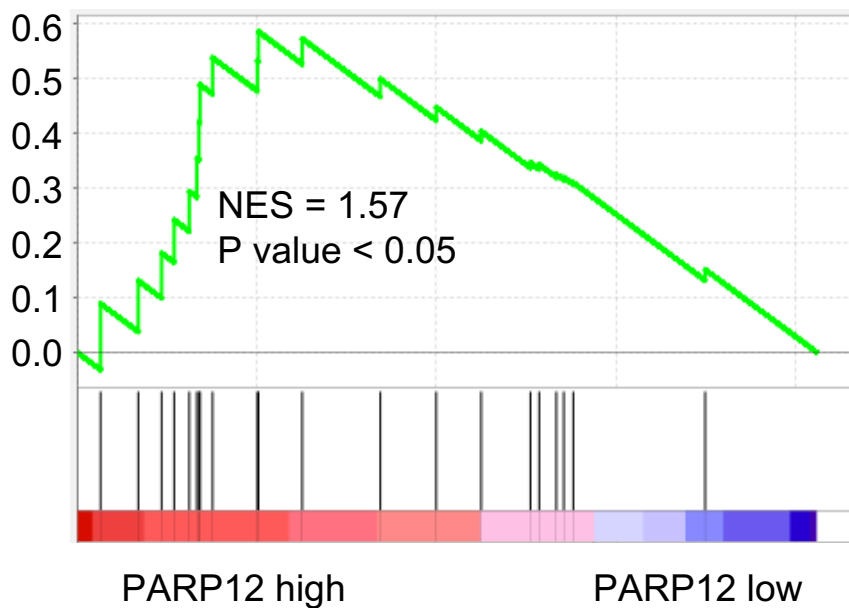

Supplement: Supplementary file 1 [file nutrients-16-02665-s001.zip › Figure S7.pdf]
